# Prevalence of HTLV-1 and HTLV-2 infection among transgender women in Brazil: A multicenter study

**DOI:** 10.1371/journal.pntd.0014581

**Published:** 2026-08-07

**Authors:** Carolina Amianti, Larissa Melo Bandeira, Regina Célia Moreira, Andréa Fachel Leal, Daniela Riva Knauth, Laio Magno, Inês Dourado, Rita Suely Bacuri de Queiroz, Katia Cristina Bassichetto, Gustavo Santa Roza Saggese, Maria Amelia de Sousa Mascena Veras, Ana Rita Coimbra Motta-Castro

**Affiliations:** 1 Universidade Federal de Mato Grosso do Sul, Campo Grande, Mato Grosso do Sul, Brazil; 2 Instituto Adolfo Lutz, Centro de Virologia, Laboratório de Hepatites Virais, São Paulo, São Paulo, Brazil; 3 Universidade Federal do Rio Grande do Sul, Departamento de Sociologia - Instituto de Filosofia e Ciências Humanas, Porto Alegre, Rio Grande do Sul, Brazil; 4 Universidade Federal do Rio Grande do Sul, Departamento de Medicina Social – Faculdade de Medicina, Porto Alegre, Rio Grande do Sul, Brazil; 5 Universidade do Estado da Bahia, Departamento de Ciências da Vida, Salvador, Bahia, Brazil; 6 Universidade Federal da Bahia, Instituto de Saúde Pública, Salvador, Bahia, Brazil; 7 Fiocruz Amazônia, Instituto Leônidas e Maria Deane, Manaus, Amazonas, Brazil; 8 Faculdade de Ciências Médicas da Santa Casa de São Paulo, São Paulo, São Paulo, Brazil; University of South Florida, UNITED STATES OF AMERICA

## Abstract

**Background:**

Transgender Women and *Travestis* (TWT) are at increased risk for sexually transmitted infections due to contexts of vulnerability related to socioeconomic and behavioral factors. However, data on the prevalence of Human T-lymphotropic viruses type 1 and 2 (HTLV-1/2) in this population remain scarce. This study aimed to investigate the seroprevalence of HTLV-1/2 infection among TWT in five Brazilian capitals.

**Methodology/Principal findings:**

A cross-sectional study was conducted from 2019 to 2021 in five Brazilian capital cities, representing all geographic regions of the country. A total of 1,269 blood samples were tested for HTLV-1/2 antibodies using ELISA and Western Blot assays. Sociodemographic and behavioral data were obtained through structured interviews. Among 1,269 samples, most participants identifying as transgender women (59.18%) or *travestis* (29.79%) with a median age of 30 years. A total of 12 samples tested positive for anti-HTLV-1/2 (0.94%; CI 95%: 0.53–1.65), with positive cases in Manaus (n = 4, 1.50%), Salvador (n = 4, 2.22%), and Porto Alegre (n = 4, 2.12%), and none in São Paulo or Campo Grande. Associated behaviors included inconsistent condom use and a history of HIV and syphilis.

**Conclusions/Significance:**

Our study highlights the presence of HTLV-1/2 infection among TWT in Brazil, with regional differences. These findings underscore the need for targeted public health interventions, including increased screening, health education, and access to healthcare services, to address the specific vulnerabilities of this population.

## Author summary

Human T-lymphotropic viruses type 1 and 2 are among the most neglected human retroviruses, even though they infect millions of people worldwide and can cause severe diseases, including neurological disorders and leukemia. In Brazil, the prevalence of the infection has been reported in several regions and population groups, but information about transgender women remains extremely scarce. This is an important gap, due to this group often face social and structural vulnerabilities, such as discrimination, economic instability, and barriers to healthcare, which may increase their risk of sexually transmitted infections. In this study, we investigated the presence of these viruses among transgender women in five Brazilian capital cities representing all five regions of the country. By analyzing blood samples from more than one thousand participants, infections were identified in several cities, although the overall prevalence was relatively low. These findings provide one of the first nationwide perspectives on these viruses among transgender women in the literature. The results highlight the importance of expanding surveillance, improving access to testing, and strengthening prevention strategies for populations that remain underrepresented in epidemiological research.

## Introduction

Human T-lymphotropic virus type 1 (HTLV-1) is a retrovirus that can cause severe diseases such as adult T-cell leukemia/lymphoma (ATL), HTLV-1-associated myelopathy (HAM), and other inflammatory diseases [1]. Despite infecting approximately 10 million people worldwide and being considered endemic in many countries, HTLV-1 remains a neglected infection. HTLV-2 is significantly less prevalent than HTLV-1, with most documented cases occurring in specific populations, including people who use injectable drugs and Indigenous people of the Americas [2]. Although the clinical manifestations of HTLV-2 are not as well established as HTLV-1, neurological manifestations as well as inflammatory conditions have been reported [3]. HTLV-1/2 is transmitted from person to person through unprotected sexual intercourse, mother-to-child, or blood transfusion from an infected individual [4].

The prevalence of HTLV-1/2 in Brazil also varies among different population groups, in addition to geographical location. Moreover, sex workers, people who use injectable drugs, Japanese immigrants, and men who have sex with men are with heightened vulnerability [5].

TWT are at increased risk for Sexually Transmitted Infections (STIs) due to contexts of vulnerabilities associated with sex work, multiple sex partners, irregular condom use, drug use, history of incarceration, and poor socioeconomic conditions [6]. In Brazil, there is limited availability of data on HTLV-1/2 infection among TWT [7]. To address this gap, this study aimed to investigate behavioral characteristics the seroprevalence of HTLV-1/2 among TWT in five Brazilian capitals.

## Methods

### Ethics statement

This study was approved by the Ethical Committee on Human Research of Santa Casa de Misericórdia de São Paulo - SCMSP under protocol number CAAE: 81043324.8.0000.5479. Written informed consent was obtained. All research was performed following relevant guidelines and regulations.

### Study group

**Fig 1:**
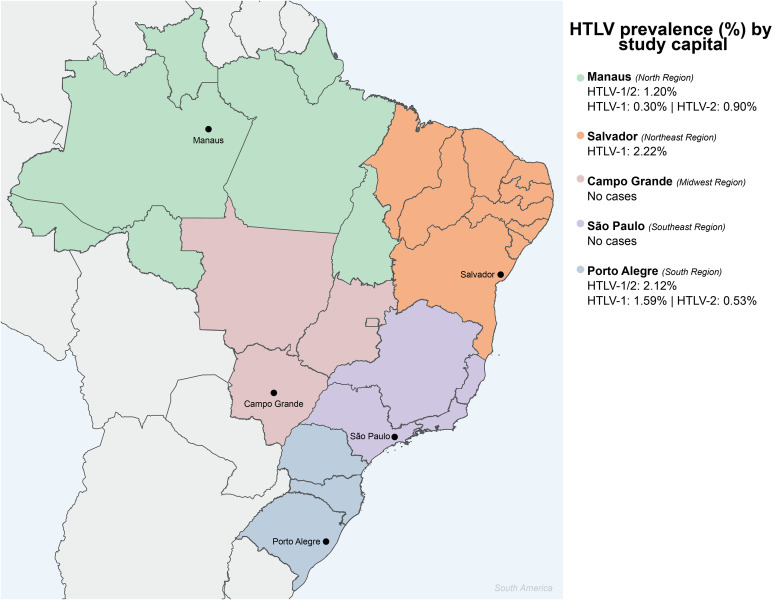
Map of Brazil showing the five geographic regions included in the study. All five regions of Brazil are highlighted in different colors, with states within the same region displayed in the same color. The capitals where the study was conducted are indicated above their respective states. Software program: QGIS 3.26.2. The basemap shapefiles used to elaborate this map: Instituto Brasileiro de Geografia e Estatística (IBGE) distributed under CC BY 4.0 license (https://biblioteca.ibge.gov.br/visualizacao/livros/liv102268.pdf). Municipal mesh dataset available at: (https://www.ibge.gov.br/en/geosciences/territorial-organization/territorial-meshes/2786-np-municipal-mesh/18890-municipal-mesh.html?lang=en-GB).

This multicentric, cross-sectional survey was conducted using information from databases and samples from the biorepository from “Study of Prevalence of Syphilis and Other STIs among Transvestites and Transgender Women in Brazil: Care and Prevention (TransOdara)” project. From December 2019 to July 2021, the five study sites: Campo Grande capital of Mato Grosso do Sul (Midwest), Manaus capital of Amazonas (North), Porto Alegre capital of Rio Grande do Sul (South), Salvador capital of Bahia (Northeast) and São Paulo capital of São Paulo (Southeast), covering all five geographic regions of the country (Fig 1).

Participants were recruited using the respondent-driven sampling (RDS) chain-referral method, which begins with “seeds”, a convenience sample of hard-to-reach individuals from the target population [8]. This sampling method has also been successfully used to recruit transgender women and *travestis* in other studies conducted in Brazil [9].

During the formative research phase at each study site, potential “seeds” (seven to nine) were selected through in-depth interviews and focus groups with members of the transgender women and *travesti* community. The criteria for selecting “seeds” encompassed connections to an extensive social network of potential participants and ensuring adequate diversity in characteristics of interest, such as educational attainment and engagement in sex work. Each seed was allocated a limited number of coupons (five to six) to distribute to other individuals within their social network, and subsequent participants were similarly provided with a comparable number of coupons to invite others [10].

The inclusion criteria were having the minimum age of 18 years, having been assigned male at birth, self-identifying as a female gender identity (transgender women, *travestis,* or other feminine identification), residing in the metropolitan area of the study sites, and presenting a valid study coupon to participate in the research. Participants underwent an interview with a standardized questionnaire containing sociodemographic and behavior information, and provided blood samples. After completing the questionnaire, participants underwent venipuncture for blood collection to perform rapid tests (RT) for HIV, syphilis, hepatitis B, and C, and provided biological samples for the detection of other STIs [6,10–12].

A minimum sample size of 1,280 (increased by 10% to account for potential losses) transgender women and *travestis* was calculated *a priori* to estimate the prevalence of active syphilis (VDRL titers >1:8) with acceptable standard errors at each study site, using an appropriate method for populations of unknown size and considering the population size of the respective cities. However, for the present study, only samples with sufficient biological sample volume available for HTLV laboratory testing were included (n = 1,269). The research employed a mixed methodology with a convergent design featuring a simultaneous bidirectional structure for data fusion analysis, which entails an interactive reflection of qualitative and quantitative perspectives, as reported by Veras et al., 2023 [10].

### Laboratory tests

The original dataset consisted of 1,317 serological samples from participants. However, samples with sufficient volume for conducting the analyses were obtained from 1,269 participants. All of them were screened using a commercial enzyme-linked immunosorbent assay (ELISA) kit (Murex HTLV I + II—DiaSorin), following the manufacturer’s instructions, aiming to detect anti-HTLV-1/2 antibodies. Positive samples were repeatedly tested and confirmed by an HTLV-1/2 Western Blot (WB) assay (MP Diagnostics HTLV BLOT 2.4, Singapore). HTLV infection was defined as positive in both the ELISA screening and confirmatory WB tests. The confirmatory test (WB) also types HTLV-1/2: HTLV-1 as reactivity to GAG (p19 with or without p24) and ENV (GD21 and rgp46-I) and HTLV-2 as reactivity to GAG (p24 with or without p19) and ENV (GD21 and rgp46-II) according to the manufacturer’s guidelines.

### Data analyses

All data were collected and managed as a single-entry using REDCap, hosted at the Santa Casa de São Paulo School of Medical Sciences. Excel was used for data cleaning and Stata software (version 13.0; Stata Corporation, College Station, TX, USA) was used for statistical analysis.

In the study, the term TWT was considered as an umbrella term that includes all individuals who self-identifying as a female gender identity (transgender women, *travesti,* or other feminine identification). Sexual orientation was self-reported by each participant during the structured interview and was classified into the following categories: asexual, heterosexual, homosexual, bisexual/pansexual, or refused to answer. In the context of TWT, gender-identify as women, the category “heterosexual” refers to participants who reported relationships with men, whereas “homosexual” refers to those who reported relationships with woman. For syphilis, infection was considered evidence of lifetime exposure to *Treponema* pallidum, as detected by serological tests.

To estimate the prevalence of HTLV-1/2, each city was treated as a cluster due to peer recruitment and network diversity. RDS weights were not used in the analysis, due to studies that have shown that it does not improve model performance and may introduce more uncertainty [13,14]. Categorical sociodemographic and behavioral variables were reported as absolute and percentage frequencies, while the continuous were described using median and range. The prevalence of anti-HTLV-1/2 was calculated with a 95% confidence interval (95% CI). Fisher’s exact test was used when 25% or more of the cells had expected frequencies lower than five; otherwise, Chi-square was applied to evaluate the sociodemographic and behavioral characteristics of HTLV-1/2 infection. *P*-values less than 0.05 were considered statistically significant.

## Results

A total of 1,269 participants were included in the study: 401 (31.60%) from São Paulo, 332 (26.16%) from Manaus, 188 (14.81%) from Porto Alegre, 180 (14.18%) from Salvador, and 168 (13.24%) from Campo Grande. The majority identified as transgender women (59.18%), followed by *travestis* (29.79%). The overall profile of the group was predominantly mixed race (43.66%) with a median age of 30 years (1IQ: 25 to IQ3: 39 years old), heterosexual (78.49%), single (70.06%), and (54.06%) completed high school.

Regarding the vulnerable conditions, only 23 (1.18%) are homeless, most of them (62.17%) reported having their own or rented housing. No more than 64 (5.04%) completed university education. History of intimate partner violence, commercial sex work and being unemployed were reported by 49.96%, 20.80% and 22.77%, respectively. The majority (51.93%) received monthly income lower than the minimum wage (approximately U$186). Most participants (84.87%) reported having experienced discrimination for being transgender; however, the majority (69.35%) had never experienced inadequate healthcare services related to gender identity, although a substantial proportion reported such experiences. Additionally, 58.23% reported no problems accessing healthcare services, and 917 (72.26%) reported having attended a medical appointment in the last 12 months. Focusing on each capital in the study, their profiles are similar to the national description above described, except in Manaus, where the majority 159 (47.89%) temporarily lives with family or friends (*P* < 0.05), a few had ever worked as sex workers 39 (11.75%) and 115 (34.64%) are unemployed (*P* < 0.05). Regarding their sociodemographic and behavioral characteristics, Table 1 summarizes them. Considering all capital cities, a borderline p-value was observed with HIV coinfection (p = 0.05), whereas a significant difference was identified for lifetime syphilis coinfection (p = 0.03). In Porto Alegre, a borderline p-value of HTLV infection was observed according to increasing age (p = 0.05), while a significant difference was found for history of crack cocaine use (p < 0.03).

**Table 1:** Sociodemographic and behavioral characteristics of participants (n = 1,269) according to anti-HTLV-1/2 antibodies, overall and by city, Brazil.

Variable	Brazil		Campo Grande	São Paulo	Manaus		Porto Alegre		Salvador	
	Negative	Positive	Negative	Negative	Negative	Positive	Negative	Positive	Negative	Positive
***Age (years)***										
<40	962 (99.28%)	7 (0.72%)	129 (100%)	281 (100%)	267 (99.26%)	2 (0.74%)	138 (99.28%)	1 (0.72%)	174 (97.35%)	4
≥40	295 (98.33%)	5 (1.67%)	39 (100%)	120 (100%)	61 (96.83%)	2 (3.17%)	46 (93.88%)	3 (6.12%)	29 (100%)	–
***Ethnicity***										
White	322 (99.38%)	2 (0.62%)	49 (100%)	106 (100%)	59 (98.33%)	1 (1.67%)	87 (98.86%)	1 (1.14%)	21 (100%)	–
Mixed race	548 (98.92%)	6 (1.08%)	71 (100%)	192 (100%)	197 (98.99%)	2 (1.01)	35 (97.22%)	1 (2.78%)	53 (94.64%)	3 (5.36%)
Black	334 (99.11%)	3 (0.89%)	37 (100%)	90 (100%)	50 (98.04%)	1 (1.96%)	59 (98.33%)	1 (1.67%)	98 (98.99%)	1 (1.01%)
Others	42 (97.67%)	1 (2.33%)	3 (100%)	12 (100%)	20 (100%)	–	3 (75.00%)	1 (25.00%)	4 (100%)	–
Refuse to answer, does now know	11 (100%)	–	8 (100%)	1 (100%)	2 (100%)	–	–	–	–	–
***Schooling***										
College education	64 (100%)	–	5 (100%)	23 (100%)	20 (100%)	–	11 (100%)	–	5 (100%)	–
High school	682 (99.42%)	4 (0.58%)	90 (100%)	218 (100%)	188 (98.95%)	2 (1.05%)	92 (98.92%)	1 (1.08%)	94 (98.95%)	1 (1.05%)
Elementary school	288 (97.96%)	6 (2.04%)	35 (100%)	94 (100%)	58 (98.31%)	1 (1.69%)	55 (96.49%)	2 (3.51%)	46 (93.88%)	3 (6.12%)
Incomplete elementary school	214 (99.07%)	2 (0.93%)	37 (100%)	65 (100%)	52 (98.31%)	1 (1.69%)	25 (96.15%)	1 (3.85%)	29 (100%)	–
Illiterate	6 (100%)	–	–	1 (100%)	3 (100%)	–	1 (100%)	–	1 (100%)	–
Refuse to answer, does now know	3 (100%)	–	1 (100%)	–	1 (100%)	–	–	–	1 (100%)	–
***Living conditions***										
Own or rented house	782 (99.11%)	7 (0.89%)	99 (100%)	290 (100%)	130 (99.24%)	1 (0.76%)	123 (97.62%)	3 (2.38%)	140 (97.90%)	3 (2.10%)
Live with family/friends	332 (99.10%)	3 (0.90%)	58 (100%)	42 ((100%)	157 (98.74%)	2 (1.26%)	49 (100%)	–	26 (96.30%)	1 (3.70%)
Boarding house	16 (100%)	–	1 (100%)	8 (100%)	–	–	3 (100%)	–	4 (100%)	–
Shelter/institution	53 (100%)	–	2 (100%)	31 (100%)	10 (100%)	–	5 (100%)	–	5 (100%)	–
Lives at work/Brothel	8 (100%)	–	2 (100%)	4 (100%)	–	–	1 (100%)	–	1 (100%)	–
Homeless	22 (95.65%)	1 (4.45%)	6 (100%	10 (100%)	11 (100%)	–	1 (50%)	1 (50%)	–	–
Refuse to answer, does now know	44 (97.78%)	1 (2.22%)	–	16 (100%)	20 (95.24%)	1 (4.76%)	2 (100%)		–	–
***Sexual orientation***										
Asexual	3 (100%)	–	1 (100%)	1 (100%)	–	–	1 (100%)	–	–	–
Heterosexual	985 (98.90%)	11 (1.10%)	98 (100%)	344 (100%)	283 (98.61%)	4 (1.39%)	121 (97.58%)	3 (2.42%)	139 (97.20%)	4 (2.80%)
Homosexual	91 (98.91%)	1 (1.09%)	32 (100%)	15 (100%)	22 (100%)	–	15 (93.75%)	1 (6.25%)	7 (100%)	–
Bisexual/pansexual	165 (100%)	–	35 (100%)	38 (100%)	21 (100%)	–	44 (100%)	–	27 (100%)	–
Refuse to answer, does now know	13 (100%)	–	2 (100%)	3 (100%)	2 (100%)	–	3 (100%)	–	3 (100%)	–
***Marital status***										
Married/In a relationship	354 (99.44%)	2 (0.56%)	35 (100%)	138 (100%)	76 (98.70%)	1 (1.30%)	56 (98.25%)	1 (1.75%)	49 (100%)	–
Single	879 (98.88%)	10 (1.12%)	125 (100%)	259 (100%)	249 (98.81%)	3 (1.19%)	121 (97.58%)	3 (2.42%)	125 (96.90%)	4 (3.10%)
Divorced/Widow	21 (100%)	–	7 (100%)	4 (100%)	2 (100%)	–	6 (100%)	–	2 (100%)	–
Refuse to answer, does now know	3 (100%)	–	1 (100%)	–	1 (100%)	–	1 (100%)	–	–	–
***Work***										
Employment with a signed contract	126 (99.21%)	1 (0.79%)	11 (100%)	68 (100%)	21 (100%)	–	17 (100%)	–	9 (90.00%)	1 (10.00%)
Informal employment	452 (99.12%)	4 (0.88%)	45 (100%)	133 (100%)	130 (98.48%)	2 (1.52%)	60 (96.77%)	2 (3.23%)	84 (100%)	–
Student	50 (100%)	–	4 (100%)	29 (100%)	3 (100%)	–	10 (100%)	–	4 (100%)	–
Retired/On benefit/Housewife	34 (97.14%)	1 (2.86%)	6 (100%)	10 (100%)	8 (100%)	–	10 (90.91%)	1 (9.09%)	–	–
Sex worker	261 (98.86%)	3 (1.14%)	52 (100%)	86 (100%)	39 (100%)	–	36 (100%)	–	48 (94.12%)	3 (5.88%)
Unemployed	286 (98.96%)	3 (1.04%)	27 (100%)	69 (100%)	113 (98.26%)	2 (1.74%)	47 (97.92%)	1 (2.08%)	3 (100%)	–
Other, refuse to answer, does now know	48 (100%)	–	23 (100%)	6 (100%)	14 (100%)	–	4 (100%)	–	1 (100%)	–
***Monthly income***										
≥ 3 minimum wages	158 (100%)	–	38 (100%)	47 (100%)	20 (100%)	–	34 (100%)	–	19 (100%)	–
1-2 minimum wages	334 (98.82%)	4 (1.18%)	62 (100%)	127 (100%)	60 (98.36%)	1 (1.64%)	49 (100%)	1 (2.00%)	36 (94.74%)	2 (5.26%)
<1 minimum wages	653 (99.09%)	6 (0.91%)	59 (100%)	214 (100%)	182 (99.45%)	1 (0.55%)	92 (96.84%)	3 (3.16%)	106 (98.15%)	2 (1.85%)
Refuse to answer, does now know	112 (98.25%)	2 (1.75%)	9 (100%)	13 (100%)	66 (97.06%)	2 (2.94%)	9 (100%)	4 (2.13%)	15 (100%)	–
***History of incarceration***										
No	967 (99.18%)	8 (0.82%)	122 (100%)	311 (100%)	247 (99.60%)	1 (0.40%)	147 (98.00%)	3 (2.00%)	140 (97.22%)	4 (2.78%)
Yes	283 (98.61%)	4 (1.39%)	45 (100%)	87 (100%)	80 (96.39%)	3 (3.61%)	36 (97.30%)	1 (2.70%)	35 (100%)	–
Refuse to answer, does now know	7 (100%)	–	1 (100%)	3 (100%)	1 (100%)	–	1 (100%)	–	1 (100%)	–
***History of crack use***										
No	1,075 (99.26%)	8 (0.74%)	131 (100%)	328 (100%)	305 (99.03%)	3 (0.97%)	144 (99.31%)	1 (0.69%)	167 (97.66%)	4 (2.34%)
Yes	180 (97.83%)	4 (2.17%)	37 (100%)	73 (100%)	22 (95.65%)	1 (4.35%)	40 (93.02%)	3 (6.98%)	8 (100%)	–
Refuse to answer, does now know	2 (100%)	–	–	–	1 (100%)	–	–	–	1 (100%)	
***History of opiate use***										
No	1,231 (99.11%)	11 (0.89%)	162 (100%)	395 (100%)	327 (98.79%)	4 (1.21%)	175 (97.77%)	4 (2.23%)	172 (98.29%)	3 (1.71%)
Yes	23 (95.83%)	1 (4.17%)	6 (100%)	6 (100%)	–	–	9 (100%)	–	6 (66.67%)	1 (33.33%)
Refuse to answer, does now know	3 (100%)	–	–	–	1 (100%)	–	–	–	2 (100%)	–
***History of sharing needles***										
No	1,216 (99.10%)	11 (0.90%)	165 (100%)	389 (100%)	313 (98.74%)	4 (1.26%)	180 (98.36%)	3 (1.64%)	169 (97.69%)	4 (2.31%)
Yes	23 (95.83%)	1 (4.17%)	2 (100%)	9 (100%)	4 (100%)	–	3 (75.00%)	1 (25.00%)	5 (100%)	–
Refuse to answer, does now know	18 (100%)	–	1 (100%)	3 (100%)	11 (100%)	–	1 (100%)	–	2 (100%)	–
***Condom use with steady partner in the last sexual contact***										
Yes	208 (100%)	–	31 (100%)	68 (100%)	45 (100%)	–	28 (100%)	–	36 (100%)	–
No	391 (98.74%)	5 (1.26%)	49 (100%)	139 (100%)	58 (98.31%)	1 (1.69%)	75 (97.40%)	2 (2.60%)	70 (97.22%)	2 (2.78%)
Refuse to answer, does now know	658 (98.95%)	7 (1.05%)	88 (100%)	194 (100%)	225 (98.68%)	3 (1.32%)	81 (97.59%)	2 (2.41%)	70 (97.22%)	2 (2.78%)
***Condom use with casual partner in the last sexual contact***										
Yes	331 (99.10%)	3 (0.90%)	44 (100%)	92 (100%)	62 (98.41%)	1 (1.59%)	77 (98.72%)	1 (1.28%)	56 (98.25%)	1 (1.75%)
No	208 (99.05%)	2 (0.95%)	34 (100%)	72 (100%)	40 (100%)	–	32 (100%)	–	30 (93.75%)	2 (6.25%)
Refuse to answer, does now know	718 (99.03%)	7 (0.97%)	90 (100%)	237 (100%)	226 (98.69%)	3 (1.31%)	75 (96.87%)	3 (3.85%)	90 (98.90%)	1 (1.10%)
***Lifetime syphilis***										
No	475 (99.79%)	1 (0.21%)	58 (100%)	159 (100%)	124 (100%)	–	67 (98.53%)	1 (1.47%)	67 (100%)	–
Yes	782 (98.61%)	11 (1.39%)	110 (100%)	242 (100%)	204 (100%)	4 (1.92%)	117 (97.50%)	3 (2.50%)	109 (96.46%)	4 (3.54%)
***Anti-HIV-1/2***										
No	822 (99.52%)	4 (0.48%)	120 (100%)	295 (100%)	209 (99.52%)	1 (0.48%)	83 (100%)	–	115 (97.46%)	3 (2.54%)
Yes	424 (98.15%)	8 (1.85%)	45 (100%)	106 (100%)	118 (97.52%)	3 (2.48%)	101 (96.19%)	4 (3.81%)	54 (98.18%)	1 (1.82%)
Sample not available	11 (100%)	–	3 (100%)	–	1 (100%)	–	–	–	7 (100%)	–

Values are presented as n (%). HTLV: Human T-Lymphotropic Virus. HIV: Human Immunodeficiency Virus. “Refuse to answer/does not know” includes participants who declined or were unsure of the information.

Of all 1,269 samples tested for anti-HTLV-1/2, 12 samples were positive for anti-HTLV-1/2, with an overall prevalence rate of 0.94% (CI 95%: 0.53 - 1.65), 0.63% (CI 95%: 0.31 - 1.25) of HTLV-1 and 0.31% (CI 95%: 0.11 - 0.83) of HTLV-2 in the study. Manaus, in the North, presented four positive samples, only one for HTLV-1, 0.30% (CI 95%: 0.04 - 2.12), and three for HTLV-2, with a prevalence rate of 0.90% (CI 95%: 0.29 - 2.77), with 1,20% (CI 95%: 0.45 - 3.18) for the type 1 and 2.

In the Northeast, Salvador, four positive samples for HTLV-1, 2.22% (CI 95%: 0.82 - 5.82%). Porto Alegre, in the South, also has four positive samples, but three cases for HTLV-1 1.59% (CI 95%: 0.51 - 4.87) and one for HTLV-2 0.53% (CI 95%: 0.07 - 3.74), with 2,12% (CI 95%: 0.79 - 5.57%) for the type 1 and 2. Campo Grande and São Paulo, Midwest and Southeast, respectively, did not perform any positive sample. Fig 1 demonstrates the prevalence in each capital in the respective state and region. Considering the risk behaviors of these participants, the majority did not use condoms in the last sexual relation (Table 2).

**Table 2:** Characteristics of all participants with positive results for anti-HTLV-1/2, Brazil (n = 12).

Variable	ID-2114	ID-2115	ID-2214	ID-2235	ID-3056	ID-3057	ID-3110	ID-3131	ID-4096	ID-4122	ID-4123	ID-4184
**City**	Manaus	Manaus	Manaus	Manaus	Porto Alegre	Porto Alegre	Porto Alegre	Porto Alegre	Salvador	Salvador	Salvador	Salvador
**Type of HTLV**	HTLV-2	HTLV-1	HTLV-2	HTLV-2	HTLV-1	HTLV-1	HTLV-2	HTLV-1	HTLV-1	HTLV-1	HTLV-1	HTLV-1
**Age (years)**	50	45	26	31	33	50	40	42	37	25	21	37
**Ethnicity**	Mixed race	Mixed race	Black	White	Black	Mixed race	White	Asian descendant	Black	Mixed race	Mixed race	Mixed race
**Sexual orientation**	Heterosexual	Heterosexual	Heterosexual	Heterosexual	Heterosexual	Heterosexual	Heterosexual	Homosexual	Heterosexual	Heterosexual	Heterosexual	Heterosexual
**Current sex work**	No	No	No	No	No	No	No	No	No	Yes	Yes	Yes
**History of incarceration**	Yes	Yes	No	Yes	No	No	Yes	No	No	No	No	No
**Condom use (steady partner) in the last sexual contact**	NA	NA	NA	No	No	No	NA	NA	NA	No	NA	No
**Condom use (casual partner) last sexual contact**	Yes	NA	NA	NA	NA	NA	Yes	NA	No	No	NA	Yes
**History of crack use**	No	No	Yes	No	Yes	No	Yes	Yes	No	No	No	No
**History of opiate use**	No	No	No	No	No	No	No	No	No	Yes	No	No
**History of sharing needles**	No	No	No	No	No	No	Yes	No	No	No	No	No
**Anti-HIV-1/2**	No	Yes	Yes	Yes	Yes	Yes	Yes	Yes	No	No	No	Yes
**Lifetime syphilis**	Yes	Yes	Yes	Yes	No	Yes	Yes	Yes	Yes	Yes	Yes	Yes

Individual-level characteristics of participants with confirmed HTLV-1/2 infection. HTLV type determined by serological assays. NA (Not applicable) indicates participants who did not respond or were unsure of the information.

## Discussion

As far as we know, this was the first multicenter nationwide study investigating HTLV-1/2 infection among TWT across all geographic regions of Brazil.

Our study found an overall network prevalence rate of 0.94% (95% CI: 0.53–1.65) for HTLV-1/2. It can be classified as low to medium prevalence when considering the confidence interval when compared with the stratification carried out by Gonçalves et al., 2010 [15]. However, when compared to the general population, a study conducted in Belém, capital of Pará State found a lower prevalence, 0.38% (CI 0.07–0.69%) [16]. In this scenario, our findings suggest that TWT may have a higher prevalence of HTLV-1/2 infections, however another study conducted with the general population in Campo Grande found a similar prevalence 0.82% (CI 95% 0.34–1.96) [17]. Therefore, specific and regional characteristics of the groups must be considered individually. The prevalence found in our study aligns with those reported by Carneiro et al. (2024), who conducted a local study among TWT in Goiás, Central Brazil [7], they reported an HTLV-1 prevalence of 1.3%, but after having weighted their data by RDSAT 1.0% (95% CI: 0.0–1.9) in their cohort.

Interestingly, no positive samples were detected in Campo Grande and São Paulo in our study, but due to the design of the study it is not possible to extrapolate that there are not any TWT positive for HTLV-1/2 infection in these sites, since the presence of the virus were already reported in both capitals [17,18].

Furthermore, were demonstrated a borderline difference between the prevalence of HTLV-1/2 infection and HIV coinfection and a significant difference with lifetime syphilis infection. The first study that reports HIV and HTLV-1/2 coinfection in Brazil found a prevalence of 7.4% [19]; other studies conducted found a prevalence of this coinfection ranging from 1.61% to 2.4% [20–22]. Individuals living with HIV may have a higher risk of HTLV infection due to a vulnerability, as both viruses share similar transmission routes [23]. The same aspect could be associated with syphilis, contributing to an increased prevalence of these coinfections [24]. Additionally, a possible reason for this increase is the presence of genital ulcers, present in syphilis infection [25].

Salvador, Bahia, stands out as the Brazilian city with the highest prevalence of HTLV-1 infection. Previous studies indicate that prevalence is higher among women, increases with age, and is more elevated among those with lower socioeconomic conditions [26]. Four HTLV-1-positive samples were identified in this city in our study; three individuals self-identified as mixed-race and one as black; none had a history of incarceration; three were sex workers; none reported crack use; all tested negative for anti-HIV-1/2 but positive for lifetime syphilis; and the youngest was 21 years old. These findings suggest that, in addition to previously identified behavioral factors, unprotected sexual activity — evidenced by syphilis coinfection — may play a crucial role in HTLV-1 transmission in the Salvador study sampling. This finding aligns with a study that identifies sexual transmission as the primary mode of virus dissemination in the region [27]. Therefore, it is imperative that local public health policies intensify prevention strategies, including promoting condom use and expanding early diagnosis, especially among vulnerable populations such as sex workers.

The results obtained from Manaus, located in the North of Brazil, identified four HTLV-positive samples, including one case of HTLV-1 and three cases of HTLV-2. Three individuals had previously been incarcerated, with one reporting crack cocaine use. All were diagnosed with lifetime syphilis, and three also tested positive for anti-HIV-1/2. These findings align with those of Pontes et al. (2020), who detected HTLV-2 in urban populations of the Brazilian Amazon region [28]. The report of drug use, incarceration, and the high prevalence of HIV suggests a heightened vulnerability to HTLV infection in urban populations of the region, as previously highlighted by Bandeira et al. 2022 [29]. Several studies have demonstrated that HTLV-2 is prevalent among indigenous groups in Brazil, particularly in the Amazon region, an area known for its high endemicity. Although none of the individuals in our study were indigenous, HTLV-2 infections have been reported in non-indigenous populations as well, notably among blood donors in urban areas of the Amazon [30].

The detection of three HTLV-1 cases and one HTLV-2 case is consistent with earlier reports highlighting the presence of HTLV in Porto Alegre, South Brazil, particularly among high-risk populations. Previous research in this geographic region has indicated that HTLV-1 is more frequently associated with neurological complications such as HAM, whereas HTLV-2 is more commonly found among Indigenous and marginalized populations, such as injection drug users [31,32].

Our findings further revealed a difference (p = 0.03) between HTLV-1/2 infection and crack cocaine use among participants from Porto Alegre. This practice has been associated with an increased risk of STIs not only due to mucosal exposure to bloodborne viruses caused by burns and pipe sharing, but also because it is frequently related to high-risk sexual behaviors, including inconsistent condom use and multiple sexual partnerships [33]. The HTLV positive individuals presented a borderline difference with increasing age (p = 0.05) which is expected, particularly more expressive from the fourth decade of life [34,35]. Also in Porto Alegre, all individuals positive for anti-HTLV antibodies were also HIV-positive, and three with syphilis, highlighting the interconnected spread of STIs in vulnerable populations. Notably, the HTLV-2-positive participant reported both crack use and injection drug use, aligning with previous studies that associate HTLV-2 transmission with drug-related behaviors [36]. Another study reported the occurrence of HAM cases in Porto Alegre, suggesting that local transmission networks may contribute to the persistence of HTLV-1 infections [37].

Transgender individuals demonstrate a high risk for depression, anxiety, low self-esteem and attempted suicide [38,39]. This finding is also reported in studies conducted specifically in transgender women, where low self-esteem, lifetime polysubstance use, and intimate partner violence have been linked to sexual risk behavior such as unprotected sex, high levels of substance use, sex under the influence of drugs or alcohol, multiple partners, and commercial sex work [40–44]. Our data did not demonstrate a difference between non-use of condoms and HTLV infection. This may be due to the homogeneity of the group, in which the majority of participants of the study either do not use condoms, opted not to disclose this information, or were unable to recall their condom use practices. This scenario creates favorable conditions for the continued transmission of the virus to other individuals.

Some limitations must be considered, particularly regarding self-reported data and the possible underreporting of sensitive behaviors due to stigma and discrimination. These factors may have limited the accuracy of some behavioral variables and, consequently, affected the interpretation of characteristics associated with HTLV infection. Regarding the RDS methodology, selection biases may occur in relation to specific profiles and biases in seed selection. Although it is a methodology frequently used in research with hard-to-reach populations. Additionally, we did not have access to clinical data beyond what was available in the database.

## Conclusions

Our findings revealed the presence of HTLV-1/2 among TWT in Brazil, with notable regional variations, social vulnerability with risk behaviors and others STIs. Despite the relatively low prevalence observed, these findings are epidemiologically relevant due to the scarcity of nationwide data on HTLV-1/2 in this population and the persistence of structural and healthcare-related vulnerabilities. The results reinforce the need for expanded surveillance and the implementation of targeted prevention and screening strategies. Furthermore, they highlight the importance of integrated public health policies that combine STI prevention, harm reduction initiatives, and equitable access to healthcare services in order to address the specific needs and vulnerabilities of TWT in Brazil.
